# Current Modalities in Soft-Tissue Reconstruction and Vascularized Adipose Engineering

**DOI:** 10.3390/biom15060780

**Published:** 2025-05-28

**Authors:** Jessica C. El-Mallah, Connie Wen, Olivia Waldron, Neekita R. Jikaria, Mohammad Hossein Asgardoon, Kevin Schlidt, Dana Goldenberg, Summer Horchler, Mary E. Landmesser, Ji Ho Park, Urara Hasegawa, Yong Wang, Dino J. Ravnic

**Affiliations:** 1 Department of Surgery, Penn State Milton S. Hershey Medical Center, 500 University Drive, Hershey, PA 17033, USA; owaldron@pennstatehealth.psu.edu (O.W.); njikaria@pennstatehealth.psu.edu (N.R.J.); masgardoon@pennstatehealth.psu.edu (M.H.A.); kschlidt@lifebridgehealth.org (K.S.); dana.goldenberg96@gmail.com (D.G.); summer.horchler@gmail.com (S.H.); mlandmesser@pennstatehealth.psu.edu (M.E.L.); jpark9@pennstatehealth.psu.edu (J.H.P.); 2 Department of Biomedical Engineering, The Pennsylvania State University, 201 Old Main, University Park, PA 16802, USA; cqw5733@psu.edu (C.W.); yxw30@psu.edu (Y.W.); 3 Department of Materials Science and Engineering, The Pennsylvania State University, 201 Old Main, University Park, PA 16802, USA; uph5002@psu.edu; 4 Huck Institutes of the Life Sciences, The Pennsylvania State University, 201 Old Main, University Park, PA 16802, USA

**Keywords:** soft-tissue reconstruction, adipose tissue engineering, biocompatible scaffolds, vascularized tissue engineering

## Abstract

Soft-tissue loss resulting from trauma or oncologic resection is a significant problem worldwide. Surgical reconstruction using adipose tissue has long been the gold-standard solution. However, these surgeries are often highly morbid, not always feasible in patients with insufficient adipose, and can have unpredictable results. Engineered soft-tissue replacements present a promising alternative. Many cell types, such as adipose-derived stem cells, have been recognized as a viable starting platform upon which new avenues in tissue engineering can be built. Additionally, efforts to develop scaffolds that can mimic the native extracellular matrix have been made with varying success. However, the suboptimal vascularization of engineered replacements is still a major limiting factor for achieving clinical translation. The current research explores the integration of all these techniques, including the use of growth factors, bioactive molecules, and advanced microsurgical techniques to enhance the vascularization process. This translational review covers the clinically standard methods of soft-tissue reconstruction and dives into emerging engineering techniques to develop vascularized adipose alternatives.

## 1. Introduction

Fat, or adipose, is a form of loose connective tissue derived from the mesoderm. It is composed mainly of adipocytes but houses a variety of cell types, including preadipocytes, stem cells, endothelial cells (ECs), pericytes, fibroblasts, macrophages, and immune cells [1,2]. Adipose is abundant and largely dispensable. Due to its ubiquitous nature, it has been used extensively for soft-tissue reconstruction throughout the body [3,4]. Unfortunately, not all individuals possess enough adipose for reconstructive applications, and engineering platforms have been developed to mitigate these insufficiencies.

### 1.1. Adipose Development

**Figure 1:**
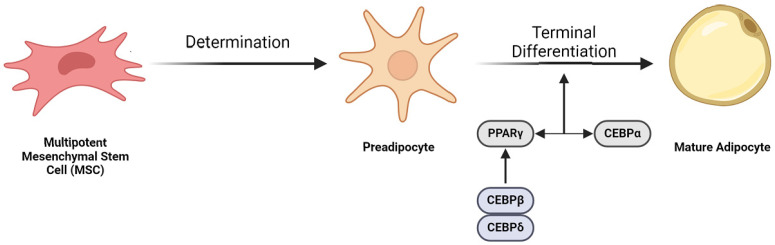
Overview of adipogenesis. MSCs serve as adipocyte precursors. The initial determination phase involves the conversion of an MSC to a preadipocyte. PPAR-γ and C/EBPα activate one another through a variety of signaling molecules and thus promote the terminal differentiation phase. The preadipocyte then undergoes terminal differentiation to a mature adipocyte. (Image created with Biorender.com).

Adipose development, or adipogenesis, is the process by which mesenchymal stem cells (MSCs) differentiate into mature adipocytes. This process is regulated by a complex transcriptional cascade. While over two dozen relevant transcription factors have been noted, PPAR-γ is the master regulator, as no other pro-adipogenic factors can function in its absence [5,6,7,8,9,10,11,12,13]. Induced by C/EBP-β and δ proteins, PPAR-γ works with C/EBP-α to establish adipocyte maturity (Figure 1) [14,15,16,17,18,19]. Understanding adipogenesis at the molecular level is central to adipose tissue engineering, as manipulating these pathways enables the directed differentiation of stem cells into functional adipocytes. This knowledge facilitates the development of biomimetic scaffolds and culture conditions that recapitulate native adipose tissue architecture and function, advancing strategies for soft-tissue reconstruction and regenerative therapies.

### 1.2. Adipose Angiogenesis

**Figure 2:**
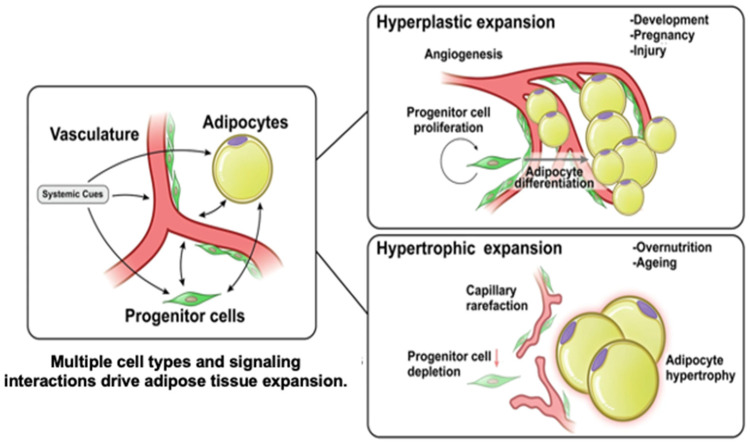
Microvascular growth in adipocyte hyperplasia vs. hypertrophy. The diagram illustrates adipose expansion with hyperplasia or hypertrophy. Adapted from Corvera et al. under the terms of the Creative Commons Attribution (CC BY) license (CC BY 4.0 Deed|Attribution 4.0 International|Creative Commons) [23].

Adipocyte angiogenesis, the formation of new blood vessels within adipose tissue in response to hypoxia and growth demands, is essential for supporting adipose expansion and function. This process is regulated by adipocyte-derived adipokines such as vascular endothelial growth factor (VEGF), which promotes neovascularization, and platelet-derived growth factor (PDGF), which contributes to vessel maturation and adipocyte development [20,21]. During adipose hyperplasia, microvascular proliferation occurs at the leading edge of the fat pat, where preadipocytes residing within the mural-cell compartment are found clustered along the expanding vasculature. This vascularization process differs from the process that takes place when adipose hypertrophy occurs (Figure 2) [22]. This spatial and functional coupling of angiogenesis and adipogenesis demonstrates the importance of vascularization in adipose tissue formation. In adipose tissue engineering, this relationship, which is leveraged by incorporating angiogenic cues and which supports vascular networks within scaffolds, is critical for sustaining cell viability, promoting integration with the host tissue, and enhancing the regenerative potential of engineered constructs.

## 2. Soft-Tissue Reconstruction

Soft-tissue loss is common with aging, traumatic injury, and oncologic resection. Over the past hundred years, surgeons have used autologous adipose grafts and flaps to correct these deficiencies. The ease of adipose harvest and its omnipresence has resulted in the vast majority of plastic surgeons utilizing these methods to treat virtually any anatomic site [24].

### 2.1. Fat-Graft Principles

Grafts lack an intrinsic vascular network, and embedded cells are reliant on diffusion from the recipient wound bed until neovascularization occurs. Because of this, only grafted adipose within a 250 µm diameter reliably survives [25]. Therefore, angiogenesis into the graft is critical and initially involves capillary inosculation from the recipient, which takes three to seven days [26]. This delay causes core necrosis, as oxygen cannot diffuse into the center of a thick graft. Consequently, this portion is dependent on intrinsic progenitor cells to induce EC transformation and capillary development [27]. Unfortunately, when the recipient site has dysfunctional microcirculation, such as in poorly controlled diabetes or irradiation, this angiogenic process is significantly impaired.

### 2.2. Fat Grafts in Clinical Care

**Figure 3:**
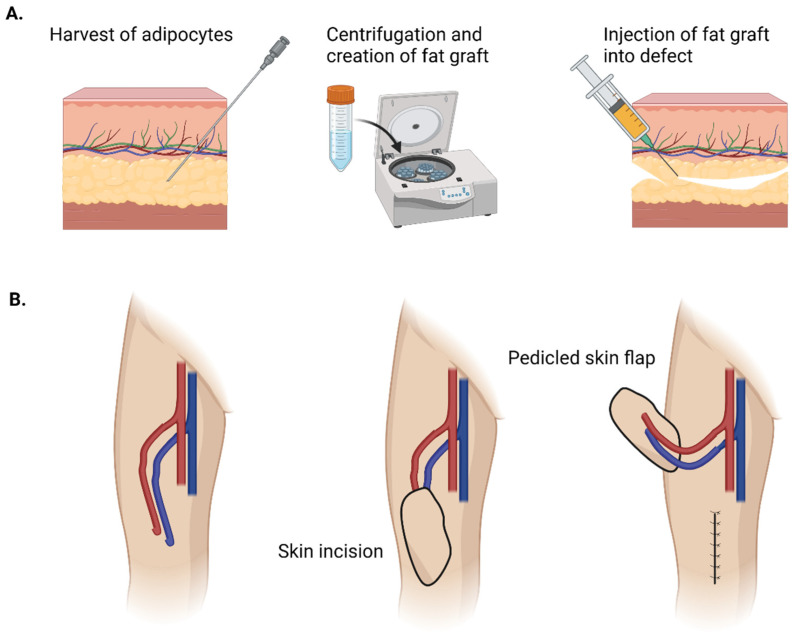
Fat grafting vs. adipose flap for soft-tissue reconstruction. (**A**) Fat-grafting technique where fat is harvested with a cannula, centrifuged, and then injected into a defect. (**B**) A pedicled flap that can be rotated into a soft-tissue defect with its accompanying vascular pedicle. (Image created with Biorender.com).

Autologous fat grafting has become an increasingly versatile tool, offering both volumetric enhancement and regenerative potential (Figure 3A). Clinically, fat grafts are commonly employed for soft-tissue augmentation in aesthetic procedures as well as in reconstructive settings such as in postmastectomy breast reconstruction, the correction of contour deformities, and the treatment of radiation-induced fibrosis [28]. The minimally invasive nature of fat grafting combined with the use of autologous tissue reduces the risk of immunogenic reactions and foreign-body responses while offering natural-appearing and durable results. Initially, there were concerns that the intrinsic adipocyte-derived stem cells (ASCs) within grafts would increase the risk of cancer propagation. However, while the relationship between fat grafting and cancer is complex, studies have demonstrated this technique’s safety and efficacy [29]. A major issue with fat grafting in breast reconstruction is that previous irradiation often requires repeated sessions of grafting. This is secondary to the detrimental effects of radiation on the microvasculature and oxygen diffusion in the recipient [30]. This highlights the importance of optimizing the recipient site prior to grafting.

### 2.3. Graft and Recipient-Site Preparation

Two fundamental factors related to adipose retention are the graft–recipient interface and interstitial-fluid pressure limitations. Due to oxygen diffusion limitations, the “viable zone” of a graft extends 200 µm from the periphery of grafted tissue, as per the cell survival theory [31]. For this reason, thin surgical cannulas have been developed to deliver fat droplets in micro-ribbon form to increase the graft–microvasculature contact area [31]. Where adequate dispersion of the microdroplets is not achieved, grafts will be prone to hypoxia and the formation of necrotic cysts. Moreover, over-grafting profoundly increases the interstitial osmotic pressure, impairing normal capillary fluid dynamics, blood flow, and oxygen delivery.

Adipocytes are also susceptible to mechanical forces. Different harvesting techniques place varying degrees of mechanical stress on fat grafts, affecting their viability and function [32]. Common harvesting modalities include hand-held syringe aspiration, suction-assisted lipectomy, and ultrasound-assisted lipectomy [33]. Several studies have investigated the effect of cannula diameter on graft survival. Unfortunately, while our understanding has been expanded, no universal approach to graft preparation and delivery has been accepted. Hence, a variety of experimental techniques have been explored [34].

Preconditioning the recipient site through external volume expansion, for instance, uses vacuum-assisted devices to enhance vascularity and the graft capacity. Khouri et al. were able to demonstrate improved graft survival rates with lower rates of appreciable necrosis in patients pre-treated with the Brava vacuum-based expander device [35,36]. However, widespread clinical adoption of this technique has been limited by the cumbersome volume expansion process, significant complication profile, and marginal long-term viable graft retention improvement [35,36].

Other innovations, including the use of alloplastic materials to stimulate local inflammation and angiogenesis, angiogenic growth factor delivery (such as VEGF or the stromal vascular fraction (SVF)), ischemic preconditioning, and microneedling have shown promise in preclinical and early clinical studies [37,38,39]. These techniques aim to optimize the recipient bed through neovascular enhancement and the reduction of fat absorption. More studies are needed to test their long-term clinical efficacy and practicality in an operative setting.

### 2.4. Adipose-Flap Principles

While fat grafting is routinely performed, surgeons and scientists continue to seek complementary and alternative options to mitigate the unpredictable results [40,41]. Specifically, due to inadequate vascularization, only around 50% of the grafted volume is maintained long-term [42,43]. This leads to patients undergoing multiple surgeries. Furthermore, fat grafting is unsuitable for voluminous defects [44,45]. For these reasons, adipose-flap surgery (Figure 3B) has become commonplace.

Flap surgery is defined as the transfer of vascularized tissue together with its feeding artery and draining vein (vascular pedicle). Since flaps carry their own blood supply, they can be of any thickness and are suitable for wound reconstruction of any depth. Autologous flaps are broadly defined as pedicled or free depending on whether their vascular pedicle remains intact or needs to be divided and reconnected to the main vascular system, respectively. Pedicled flaps, therefore, are only suitable for wounds that exist in proximity to their donor site. Because the vascularized adipose is being moved a short distance without any disturbance in blood perfusion, the approach is technically easy. Free flaps allow for the movement of tissue from further distances; however, this approach is technically challenging, requiring specialized expertise and equipment that not all centers can provide. Furthermore, complications with microsurgical reconstruction are common and include the devastating loss of a flap secondary to thrombosis (up to 10%) as well as donor-site injuries such as scarring, wound dehiscence, seromas, hernias, muscle weakness, and paresthesia [46,47,48,49,50,51]. These inherent problems lead to patient frustration, re-operation, increased morbidity, and significantly higher costs [52,53]. This has led to the emergence of engineered alternatives.

## 3. Adipose Engineering

The goal of tissue engineering is to assemble functional constructs that restore, maintain, or improve damaged tissues or whole organs. Fung introduced the term in 1985, and the seminal paper was published in 1993 by Langer and Vacanti [54]. Evolved from the field of biomaterials, it refers to the practice of combining cells, scaffolds (artificial ECMs), and biologically active molecules into functional tissues. The methodologies and combinations available have grown exponentially over the past three decades. However, translatable scale-up has been largely prevented by the issue of vascularization. Developing vascularized adipose tissue (VAT) is especially complex because it requires cell sources that support both adipogenesis and angiogenesis [55]. Vascularized adipose engineering would be a welcome addition to the surgeon’s armamentarium for soft-tissue reconstruction and be of significant benefit to patients. Various cell types can be combined with scaffolds and growth factors for vascularized adipose engineering (Table 1).

### 3.1. Stem Cell Applications in Adipose Engineering

Stem cells have gained significant traction in regenerative medicine for their ability to self-renew and to differentiate into multiple cell lineages. Because mature adipocytes are terminally differentiated and mechanically fragile, they are a poor option for VAT bioengineering. MSCs—adult stem cells isolated from various types of tissue, including skeletal muscle, peripheral blood, dermis, synovial membrane, and adipose—are a better source [56].

Among the most widely utilized stem cell types are adipose-derived stem cells (ASCs), which are easily harvested in large quantities and which display robust adipogenic and angiogenic potential [57,58]. ASCs have been incorporated into various biomaterials, including hydrogels, electrospun scaffolds, and decellularized matrices, to support tissue regeneration [59,60,61,62,63]. They secrete pro-angiogenic factors as well as extracellular vesicles to further promote vascular ingrowth [64]. The ASC donor site influences the cellular yield, with subcutaneous depots such as the thigh providing higher ASC counts and superior adipogenic potential than the abdomen, waist, or inner knee, for example [65].

Induced pluripotent stem cells (iPSCs) are stem cells obtained from somatic cells through the ectopic expression of pluripotency transcription factors that have characteristics similar to those of embryonic stem cells (ESCs) [66]. They offer a promising avenue for regenerative medicine and disease modeling as they bypass the ethical concerns associated with human ESCs. iPSCs can be produced in large numbers and directed to differentiate into vascular lineages, providing a scalable source for engineering perfusable tissue constructs [67]. While its autologous use remains limited by cost and logistical hurdles, the development of HLA-matched iPSC lines holds promise for off-the-shelf applications. These cells hold the potential to address challenges associated with immunogenic rejection and the substantial quantity of autologous ECs needed to populate a pre-vascularized scaffold for clinical use [68].

To vascularize engineered adipose tissue, ECs are frequently co-cultured with ASCs or iPSCs. Human umbilical vein endothelial cells (HUVECs) are a commonly utilized cell type in VAT engineering. These cells can be readily obtained from umbilical cords, making them a convenient and abundant cell source for in vitro studies, and they can form functional vascular networks to support angiogenesis and tissue perfusion in engineered adipose constructs [69,70,71]. When seeded alongside ASCs within decellularized scaffolds, HUVECs support vessel-like structure formation and have demonstrated effective integration following implantation in small-animal models [72]. While HUVECs are often used for in vitro studies, their clinical translation is limited due to their potential for immunogenicity [73]. Additionally, maintaining the HUVEC phenotype and functionality over extended culture periods is challenging given the high rate of apoptosis following multiple cell-culture passages [74].

Possibly more clinically translatable EC sources include endothelial progenitor cells (EPCs) and human adipose microvascular endothelial cells (hAMECs). EPCs, isolated from peripheral blood, have been co-cultured with ASCs to form microvascular networks within collagen and dermal scaffolds [75]. hAMECs, although only a small fraction of the SVF, can be enriched and used to create complex vascular networks when paired with ASCs. These cells have demonstrated synergistic effects, including more mature vessel formation and improved scaffold integration in vivo. Scaffold-free models using hAMECs and stem cells have also shown promising outcomes, highlighting a path toward fully human, immunologically compatible adipose tissue constructs suitable for reconstructive applications [76].

### 3.2. Scaffolds

The ECM is a major component of native tissues. It provides cells with mechanical and structural support through networks of collagen, reticular and elastin fibers, and glycosaminoglycans (GAGs) [77]. Cells attach to the ECM by interacting with receptors such as integrin receptors [78]. Moreover, the ECM serves as a reservoir for sequestering and releasing growth factors and signaling molecules that affect cell proliferation, differentiation, and other cellular activities through signal transduction. To recapitulate these natural ECM functions, extensive studies have been conducted to engineer scaffolds from different materials such as biopolymers, synthetic polymers, and decellularized ECM (dECM).

#### 3.2.1. Biopolymer-Based Scaffolds

Biopolymers are proteins or polysaccharides that are derived from animals or plants. These materials generally degrade enzymatically or hydrolytically and have low toxicity in the human body. In addition, many biopolymers, especially those produced in mammals, contain bioactive motifs that can interact with cells and growth factors to enhance cellular attachment and proliferation. Thus far, different biopolymers have been used for adipose tissue engineering, including collagen, gelatin, fibrin gel, and alginate.

Collagen is the most widely used biopolymer for engineering cell scaffolds. Generally, collagen scaffolds are prepared by lyophilizing acidic collagen solutions. These scaffolds have sponge-like structures with interconnected pores that are suited for cell penetration and oxygen delivery. One of the challenges in using collagen sponges is their poor mechanical strength compared with natural ECM and their uncontrolled degradation rate. One way to address these limitations is to crosslink collagen fibers chemically via crosslinking agents such as glutaraldehyde, genipin, and hexamethylene diisocyanate [79]. Kimura and colleagues reported that glutaraldehyde-crosslinked collagen sponges incorporated with preadipocytes and FGF could support fat tissue formation [80,81]. Despite the promise of this approach, crosslinking agents are generally too short to bridge collagen fibers, resulting in low crosslinking efficiency and, therefore, insufficient mechanical properties. In addition, the potential toxicity of residual crosslinking agents poses a concern [82]. To address these issues, biopolymer-based crosslinkers have been used. Davidenko et al. crosslinked collagen sponges with hyaluronic acid (HA) via the carbodiimide/*N*-hydroxysuccinimide coupling reaction, which significantly improved the dissolution resistance of the collagen sponge [83]. Zhu et al. used the same chemistry to develop porous collagen–chitosan scaffolds [84]. The combination of collagen and chitosan improved the mechanical properties of the scaffold. It was also demonstrated that the porous collagen–chitosan scaffold promoted the adhesion and proliferation of ASCs and maintained cell pluripotency [84].

Gelatin, obtained by the partial hydrolysis of collagen, is another important biopolymer in adipose tissue engineering. It maintains many of the biological functions of collagen as it contains the Arg-Gly-Asp (RGD) cell-adhesion peptide motif as well as matrix metallopeptidase (MMP)-sensitive sequences. Unlike collagen, it is soluble in water at temperatures above 30–35 °C, allowing for the facile modification of gelatin with different functional groups. Among its derivatives, gelatin methacrylamide (GelMA) has frequently been used to prepare hydrogels. GelMA can be crosslinked in the presence of a photoinitiator upon UV light irradiation. Due to the relatively mild reaction conditions, the photopolymerization of GelMA can be performed in the presence of cells, enabling their encapsulation within the hydrogel network. It has been reported that both ASCs and mature adipocytes can be encapsulated in GelMA hydrogels without affecting cell viability [85]. In addition, vascularized adipose tissue-like constructs can be generated by co-culturing ASCs and HUVECs within a GelMA hydrogel [86]. While GelMA is a promising material, phototoxicity induced by UV exposure can be a potential issue for practical applications. For this reason, other crosslinking chemistries have also been exploited to engineer gelatin-based hydrogels. For example, maleimide-functionalized gelatin (GelMAL) has been crosslinked with a dithiol crosslinker via a Michael-type addition reaction, which does not require photoinitiation [87]. It was shown that hematopoietic stem cells encapsulated in a GelMAL hydrogel generated a much lower level of reactive oxygen species (ROS) compared with those in a GelMA hydrogel, indicating that the Michael-type addition is better suited for encapsulation of cells in hydrogels because of the reduction in cell damage by ROS compared with UV-light-initiated photopolymerization. Furthermore, like collagen, gelatin contains amino and carboxyl groups that can be used for crosslinking reactions with other biopolymers. For example, gelatin was reacted with HA via the carbodiimide coupling reaction followed by a cryogelation process to fabricate a porous scaffold [88]. This scaffold exhibited mechanical properties similar to those of adipose tissue and stimulated the adipogenesis of ASCs seeded in the scaffold.

Fibrin gel is a mesh-like fibrous protein network found in blood clots. This material can be prepared by mixing fibrinogen and thrombin. Fibrin gel can bind to different growth factors as well as fibronectin, and it is degraded enzymatically by the action of plasmin [89]. These biological functions make fibrin gel an attractive biomaterial for tissue engineering. Wittman et al. demonstrated that a fibrin gel containing cells from the SVF formed vascularized adipose tissue in vivo [90]. Fibrin gels were also used to co-culture ASCs and ECs derived from peripheral blood, which led to vessel-like structure formation within the hydrogel [91]. While fibrin gel holds promise, it shows relatively fast degradation (generally, within a few days in the body), hampering its long-term applications [92]. This issue can be addressed by combining fibrin gel with other biopolymers. For example, a composite of fibrin gel and collagen microfibers was used to generate a pre-vascularized adipose tissue construct from ASCs and HUVECs. This tissue construct maintained its volume with a high cell-survival rate over three months after subcutaneous implantation [93].

Alginate is an anionic polysaccharide obtained from brown seaweed. Due to its mild gelation condition, which only requires the addition of divalent cations such as Ca^2+^, this natural polymer has been used in many biomedical applications, including wound dressing and as a cell carrier [94]. Unlike protein-based biopolymers, alginate does not contain any cell-adhesion motifs. Therefore, conjugation of functional groups such as RGD peptides is often required to support cell attachment and growth [95]. Yoo et al. homogenously mixed adipose tissues with ionically crosslinked alginate gels to generate an alginate–fat scaffold. The adipose tissue within the alginate–fat scaffold remained viable and secreted adipokines in vitro. More importantly, the alginate–fat scaffold preserved the volume of adipose tissue in vivo [96]. Since alginate is practically non-degradable in the body, alginate hydrogels are quite stable. While these gels dissociate gradually by releasing Ca^2+^ ions, dissociation rates are generally slow, which prevents cell migration and vascularization. To make alginate gels susceptible to hydrolysis, partially oxidized alginate has been developed. Kim et al. reported that in vivo injection of oxidized alginate hydrogel containing pre-differentiated human ASCs resulted in the formation of adipose tissue within ten weeks [97].

#### 3.2.2. Synthetic Scaffolds

While biopolymers have been used extensively in tissue engineering due to their bioactivity, biocompatibility, and degradability, some of their drawbacks include difficulties in fine-tuning material properties such as mechanical strength, viscoelasticity, biodegradability; high costs; batch-to-batch variability; and immunogenicity. In addition, animal-derived materials have the potential risk of transmitting infectious diseases. In contrast, synthetic polymers can be manufactured reproducibly and tailored for specific applications to fulfill the required material properties. In general, synthetic materials are bio-inert, and introducing bioactive motifs is often required to ensure sufficient cell ingrowth. Thus far, various synthetic polymers have been used as scaffold materials in adipose tissue engineering, including polyethylene glycol (PEG) and its derivatives, as well as biodegradable plastics such as polyglycolic acid (PGA) and poly(lactic-co-glycolic) acid (PLGA).

PEG is a highly water-soluble polymer commonly used for engineering drug–polymer conjugates, surface coating biomedical devices, and in scaffolds for tissue engineering. Generally, PEG polymers are chemically crosslinked to generate a hydrated 3D-network structure (hydrogel). Brandl et al. reported that enzymatically degradable PEG hydrogels can promote adipogenesis in 3T3-L1 preadipocytes [98]. To confer biodegradability and cell-adhesion capability, a collagenase-sensitive peptide sequence, as well as an integrin-binding motif, was incorporated into the PEG hydrogel network structure. It was found that the PEG hydrogels containing these peptide sequences enhanced lipid synthesis from differentiating adipocytes. One of the drawbacks of chemically crosslinked PEG hydrogels is the potential toxicity of the residual reactive functional groups within a PEG hydrogel network reacting with surrounding tissues. In addition, these systems generally require complicated administration procedures such as light irradiation and the mixing of two or more components [99]. To circumvent these issues, researchers have explored the use of thermally induced gelling systems (thermogels) based on PEG-based amphiphilic block copolymers, which are liquid at room temperature but which transform into hydrogels at body temperature [100]. Because of the unique gelling mechanism using body heat to induce a sol–gel transition without the need for additional toxic chemicals, thermogels have great potential in tissue engineering applications. Vashi et al. demonstrated that PEG–polypropylene oxide–PEG amphiphilic triblock copolymers (Pluronic F127) mixed with type I collagen could serve as an injectable scaffold for supporting adipogenic differentiation of bone marrow-derived MSCs [101].

**Figure 4:**
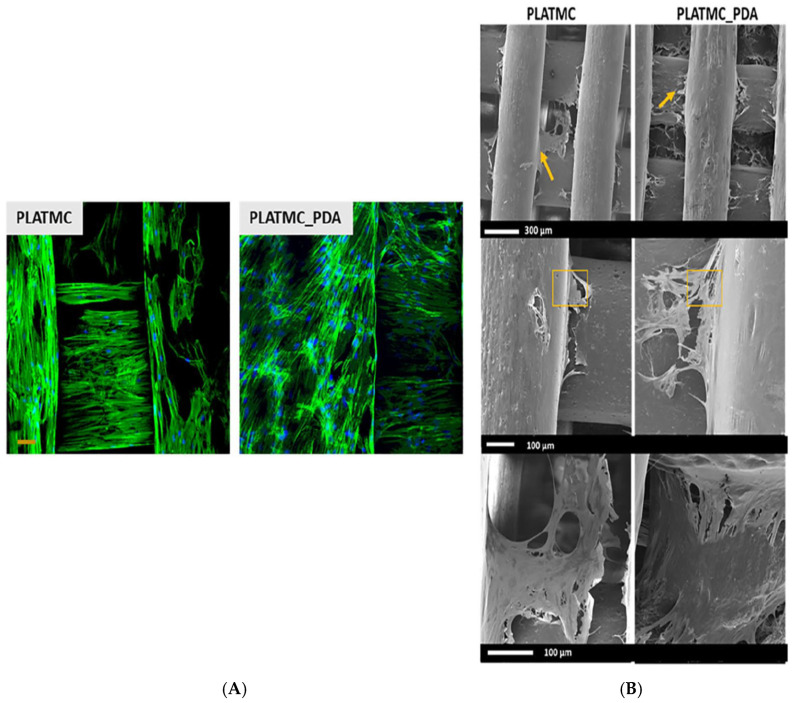
ASC response to PLATMC 3D-printed scaffolds with or without PDA coating. (**A**) Confocal images demonstrating augmented ASC distribution with the actin scaffold depicted in green and nuclei staining in blue, scale bar 50 µm. (**B**) Scanning electron microscopy images demonstrating the cell protrusion and distribution of ASCs along the PLATMC scaffolds [107]. Adapted from Jain et al. under the terms of the Creative Commons Attribution (CC BY) license (CC BY 4.0 Deed|Attribution 4.0 International|Creative Commons).

Aliphatic polyesters, such as PGA and PLGA, are semi-crystalline/glassy polymers that degrade upon hydrolysis of their ester linkages. Due to their biocompatibility, these polymers are a popular material choice in tissue engineering. In addition, these polymers have excellent processability, allowing the fabrication of different sizes and shapes of scaffolds using common manufacturing techniques such as electrospinning and 3D printing. Weiser et al. used PGA fiber meshes to culture 3T3-L1 adipocytes under adipogenic conditions [102]. Subcutaneous implantation of these cell–PGA mesh constructs led to the formation of vascularized mature adipose tissues in vivo. Xu et al. reported that the implantation of a porous PLGA scaffold seeded with ASCs in a laminectomy defect resulted in the restoration of epidural fat without scar tissue formation [103]. Additionally, Patrick et al. conducted in vivo studies using preadipocyte-seeded PLGA scaffolds in rats with successful adipose tissue development [104]. PLGA scaffolds have also demonstrated successful fat regeneration in a rabbit model. However, these are often prone to a foreign-body response, with complications such as fibrous encapsulation and inflammatory reactions. Aliphatic polycarbonates are another class of material that is of interest to biomedical engineers as these materials are degradable and resorbable [105]. Poly(trimethylene carbonate) (PTMC) is one such material that is being explored [106]. PTMC is a flexible, non-toxic scaffold that does not form acidic degradation products. Jain et al. used a 3D-printing technique to fabricate a scaffold made of poly(L-lactide-co-trimethylene carbonate) (PLATMC), which was further coated with polydopamine (PDA) for increased hydrophilicity. This scaffold augmented ASC proliferation and differentiation (Figure 4) [107].

For the successful formation of tissue-like constructs, an appropriate scaffold design is critical. Porosity is an important factor for allowing efficient cell ingrowth, sufficient nutrient and oxygen supply, and waste elimination. Pore size and interconnectivity have a significant influence on angiogenesis. It has been reported that large pores of 50–150 μm permitted mature vascularized tissue formation throughout the scaffold [108]. In addition, mechanical compatibility, degradability, and biological functionalities of scaffolds can affect adipose tissue formation.

#### 3.2.3. Decellularized ECM

Decellularized ECM (dECM) has been used in tissue engineering. The goal of decellularization is to remove all immunogenic components, such as nucleic acids, while retaining biologically active components of the ECM to provide a microenvironment for stem cell growth and differentiation after transplantation. The process of decellularization involves treating tissues with high concentrations of salts, enzymes such as trypsin, and non-ionic detergents like Triton X-100.

Adipose-tissue-derived decellularized extracellular matrix (DAM) in combination with a scaffold can be used to induce the development of adipose tissue and capillary formation [109]. DAM can be extracted from wasted adipose tissue and is composed of ECM components such as collagen, laminin, fibronectin, elastin, GAGs, and other biologically active macromolecules [110]. The fibrillar collagen and glycoproteins within the DAM provide structural stretch resistance and resilience, allowing for the dynamic remodeling of stem cells. It also contains growth factors such as VEGF, bFGF, and TGF-B, which play an important role in soft-tissue regeneration [63]. Stem cells can be seeded on the DAM and injected or transplanted into subcutaneous tissue to promote adipogenesis and angiogenesis [111]. Following co-culture of a DAM with ASCs, the DAM was demonstrated to express the adipogenic markers PPAR-γ and C/eBP-α [112]. Cell-tracking techniques have verified that this ASC/DAM combination promotes adipogenesis originating from the host [113]. These results have also been confirmed in vitro, with increased regeneration of adipocytes within DAM constructs, and further studies have confirmed the biocompatibility of the DAM with surrounding tissues [114,115,116]. Wang et al. used decellularized human adipose tissues and processed them into an injectable hydrogel for seeding with human ASCs [111]. The viability and proliferation of the ASCs were confirmed in vitro. The in vivo results showed that the dECM stimulated host-cell infiltration and neovascularization, accompanied by the formation of new adipose tissue, demonstrating the feasibility of applying this system to adipose tissue engineering. Notably, the ASC-seeded dECM did not elicit an immunogenic response [111].

The availability of adipose tissue can limit decellularization. Thus, decellularization of other tissues (i.e., placental tissue) has also been investigated for adipose tissue engineering. Flynn et al. perfused the placenta with different formulations of detergent solutions and treated it with enzymatic digestion [117]. The decellularized placenta preserved the original architecture and vascular network, and histological and immunohistochemical analyses demonstrated the successful removal of immunogenic cellular components. The ASCs attached to the decellularized placental ECM, suggesting that other types of tissues can be decellularized for adipose tissue engineering. However, dECMs have limitations related to their mechanical properties, degradation kinetics, and suboptimal cellular environments and the time-consuming nature of constructing these matrices.

#### 3.2.4. Adipose Collagen Fragments

Although acellular dermal matrices provide a framework, the decellularization process eliminates adipokines. To combat this, Xu et al. utilized adipose collagen fragments (ACFs) to capture adipokines and to functionalize these molecules to an acellular adipose matrix. Through this model, they identified the differentiation abilities of adipokines on human ASCs by evaluating the structure of neo-adipocytes and neo-adipose tissue. The adipose collagen fragments contained a diverse set of adipokines and were rich in angiogenic proteins that were able to create mature, functional, and highly vascularized adipose tissue when released in the presence of acellular adipose or dermal matrices [118].

### 3.3. Growth Factors/Biologics

Upon transplantation, the graft experiences a hostile hypoxic environment that induces growth factor and cytokine secretions that influence the newly grafted preadipocytes, adipocytes, and ASCs, as well as surrounding native adipocytes, to engage [119]. Methods have been devised to enhance and optimize this natural process by selecting specific growth factors to introduce into cell cultures, scaffolds, or grafts to promote viability. Recent advancements have utilized placental membranes to extract growth factors to create conditioned cell culture media. These membranes have abundant growth factors, including PDGF, FGF, epidermal growth factor (EGF), keratinocyte growth factor (KGF), PIGF, interleukin-4 (IL-4), transforming growth factor (TGF-β), VEGF, and tissue inhibitor metalloproteinases (TIMPs) [120]. Magana et al. found that in the presence of such factors, preadipocytes had higher cell viability in hypoxic environments when compared with normal conditions, indicating a synergistic effect between the two. Further analysis identified higher expression of VEGF-A after seven days in the hypoxic environment, suggesting these growth factors trigger angiogenesis under hypoxic conditions [121].

Furthermore, growth factors that have been paired with biodegradable scaffolds and strategies to augment their slow and controlled release from scaffolds have been extensively studied [122]. Some techniques include the use of heparin and fibronectin-binding domains to augment scaffold degradation kinetics, scaffold layering, covalent linking, and encapsulation [123]. Song et al. used decellularized adipose tissue crosslinked with heparin to encapsulate VEGF for controlled release. They found improved tissue vascularization with the benefit of a biocompatible and stable scaffold in vitro [124]. Other approaches utilize the layer-by-layer technique, alternating scaffold polymers with VEGF to allow for sequential delivery. Khanna et al. developed a polycaprolactone (PCL) scaffold with alternating layers of heparin, VEGF, and MMP-2s. The early release of VEGF followed by ECM degradation by the MMPs and heparin release improved long-term graft integration by reducing thrombogenesis [123]. Similarly, researchers used acellular adipose matrices functionalized with specific adipose-derived growth factors, including VEGF, HGF, and stromal cell-derived factor-1 (SDF-1), to induce angiogenic potential [125].

#### 3.3.1. Extracellular Vesicles

EVs are lipid-bound vesicles naturally secreted by cells that contain proteins, lipids, and nucleic acids for intercellular communication. Within VAT engineering, EVs have gained popularity for their likely role in angiogenesis [126,127]. Additionally, these molecules pose low risk for immune rejection. Studies have demonstrated that ASC-derived EVs can promote fat-graft survival through the enhancement of angiogenesis in addition to increasing graft volume retention [128,129,130]. They are also useful in the repair and regeneration of tissues but are quickly degraded. Consequently, researchers have paired them with hydrogel scaffolds for targeted delivery [131].

#### 3.3.2. Platelet-Rich Plasma

Other cell types can also augment scaffolds and serve similar functions to growth factors. A systematic review by Vyas et al. recognized platelet-rich plasma (PRP) and ASCs to be the most efficacious in promoting graft survival in vivo [132]. Li et al. demonstrated that the combination of ASCs and PRP not only promoted graft survival but maintained tissue volume in mice [133]. Sasaki et al. demonstrated a statistically significant difference between mean graft retention with PRP supplementation in fat grafting for anterior mid-face grafts compared with fat alone [134]. Furthermore, Gentile et al. demonstrated maintenance of tissue volume and shape in breast reconstruction with the addition of PRP to autologous fat transfers [135].

### 3.4. Approaches to Engineering Vascularized Adipose Tissue

Two principal approaches in VAT engineering have emerged: top–down and bottom–up [136]. The top–down approach involves seeding cells onto porous scaffolds, stimulating cell proliferation with growth factors, and cultivating the construct in a supportive environment [137,138]. The bottom–up approach (modular) utilizes individual cells or cell agglomerates, such as spheroids, organoids, and cell sheets, which are then assembled into a more complex structure to mimic native tissue [139]. This approach offers greater control over the tissue architecture.

#### 3.4.1. Top–Down Approach

In the top–down approach, 3D pre-shaped constructs can be seeded with ASCs and ECs to form mature VAT [140]. Additionally, scaffolds can deliver complementary growth factors and biologics that support VAT. Zhang et al. demonstrated that when scaffolds were integrated with human ASCs as well as microspheres that release VEGF, the constructs showed neovascularization and persistent adipose tissue and ECM formation in rats [141]. Additionally, there has been growing interest in the use of nanotechnology in vascularized adipose engineering. Nanotechnology refers to the use of nano-sized particles (drugs, proteins, etc.) that can be placed within or around scaffolds or other implantable materials to deliver molecules. These particles increase the surface area, allowing for a more widespread therapeutic effect, and can be targeted to specific tissues. The top–down approach poses many limitations, including slow vascularization, diffusion limitations, low cell densities, and a non-uniform cell distribution. For this reason, many researchers have started focusing on a bottom–up approach instead.

#### 3.4.2. Bottom–Up Approach

Modular engineering often utilizes cell clusters such as spheroids or organoids. Spheroids are a 3D cell cluster formed by exposing cells to a non-adherent environment. Spheroids exhibit close cell compaction, creating oxygen and nutrient gradients similar to that in natural tissues, thus providing the ability to mimic in vivo conditions [142]. ASCs have been cultured in 3D spheroids with success [143]. Similarly to fat grafts, spheroids can only be grown to up to 400 µm in diameter, as an increased size results in limited diffusion, leading to a necrotic core. However, recent research has shown them to have some pro-angiogenic qualities, and they are also amenable to be used as a bioink, allowing for controlled placement to maximize oxygen diffusion and vascularization [144]. Challenges persist, including achieving the ideal spheroid size and compactness, the capacity for fusion, and the high cell density required for mimicking native tissues.

Organoids, or “mini-organs”, are self-organizing in vitro cell cultures that differentiate into functional cell types with the ability to grow in a 3D environment. Generated using ESCs, iPSCs, or adult stem cells, organoids can mimic any tissue [145]. There have been several studies using ASCs to create organoids through adipogenesis and vascularization [146]. Strobel et al. aimed to incorporate vascular structures into adipose organoids by differentiating human MSCs into preadipocytes and mixing them with microvessels [147]. However, like spheroids, they suffer from oxygen diffusion limitations and slow vascular inosculation to the recipient.

### 3.5. Initiating Perfusion

Despite some success in VAT engineering, the same problem that plagues autologous fat grafts persists—the inability to provide prompt oxygen delivery upon implantation. The microcirculation within VAT needs to integrate with that of the recipient as quickly as possible to prevent necrosis. Essentially, the recipient macrovasculature needs to establish continuity with the embedded adipose microvasculature. There have been some promising advances in microsurgery to improve perfusion.

#### 3.5.1. Arteriovenous Loops

**Figure 5:**
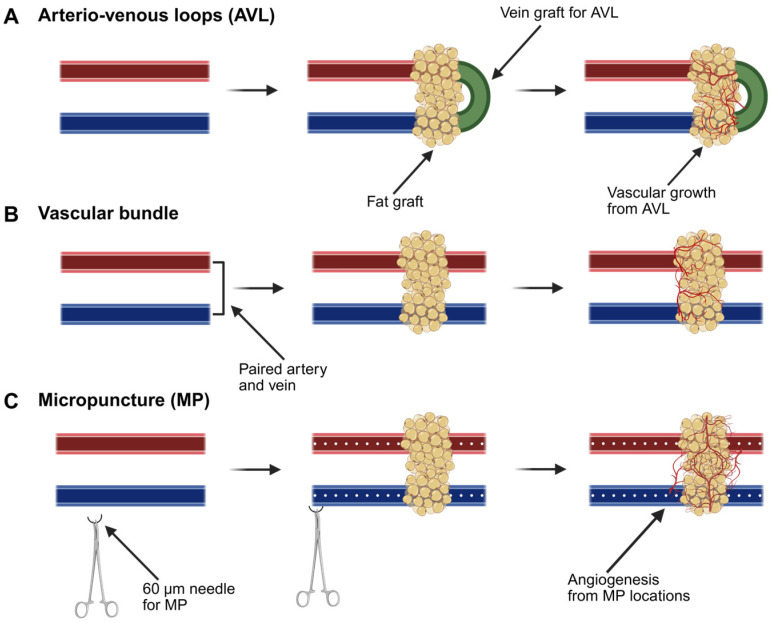
Schematic of microsurgical techniques to improve vascularization. (**A**) An AVL with a fat graft demonstrating the vascularization from a venous graft. (**B**) Fat-graft deposition over a vascular bundle showing microvascular formation. (**C**) Transmural MPs into a vascular bundle followed by placement of a fat graft demonstrating augmented vascularization. Image created with biorender.com.

One technique described extensively in the literature to promote recipient-site angiogenesis is the use of arteriovenous loops (AVLs) (Figure 5A). AVLs are deliberate arterio-venous fistulas that are created with a grafted venous interposition. AVLs can stimulate capillary formation into the surrounding matrix. It has been demonstrated that AVLs induce angiogenesis by modifying blood flow dynamics; increased flow in the AVL causes higher wall shear stress leading to the formation of new vessels [148,149]. Further analysis demonstrated that elevated shear stress leads to angiogenesis by inhibiting the klf2 gene, which is responsible for the endothelial to mesenchymal transition, and increasing the expression of pro-inflammatory, pro-angiogenic macrophages and connexin 43 (a gap-junction protein), which is typically negligible in veins [150,151]. Despite their technical complexity, AVLs have been safely utilized in plastic surgery for vascular reconstruction and subsequent flap transfer with good clinical outcomes [152]. By combining an AVL with a fat graft, the fat receives a pedicled blood supply, and angiogenesis into the graft is stimulated. The vascularized tissue can then be either left in situ or transplanted to a distal site for soft-tissue reconstruction. This model thereby mimics an autologous flap. Debels et al. combined fat grafts with an AVL in an isolation chamber and demonstrated vascularized adipogenesis [153]. The adipocytes that remained within the isolation chamber appeared to be the product of adipogenesis rather than adipocyte survival, suggesting that the success of fat grafts may be based on new adipocyte development. Similarly, Henn et al. combined an injectable nanofiber hydrogel with an AVL to engineer a soft-tissue flap, demonstrating adipose vascularization within the isolation chamber [154]. This hydrogel combination could offer an off-the-shelf injectable scaffold that produces a flap with biomechanical properties similar to that of human fat. However, AVLs are technically cumbersome to perform, and simpler microsurgical approaches have been trialed as well.

#### 3.5.2. Vascular Bundles

Another avenue for engineering VAT is with its own vascular pedicle. Here, fat grafts are placed in direct continuity with an underlying arterial and venous macrovasculature (Figure 5B). Vascular bundles are simple and effective techniques for tissue or construct vascularization. Previous studies have demonstrated the pro-angiogenic effects of vascular bundles with and without anastomoses in silk-scaffold vascularization [155]. Furthermore, Tanaka et al. demonstrated adipose tissue growth utilizing this technique in vivo [156,157]. In this study, the groins of rabbits were implanted with a tissue-growth chamber containing a vascular pedicle bundle with a collagen sponge, PRP, and bFGF. At the 12-week timepoint, adequate vascular tissue had developed sufficiently to transfer this tissue as an adipose flap with the vascular pedicle outside of the chamber [157]. A technique like this would offer the ability to spontaneously generate an autologous adipose flap for transfer without the donor-site morbidity that is currently encountered with flap tissue. Lu et al. further expanded on this model by utilizing an adipose tissue extract in combination with the chamber model to further promote tissue growth and vascularization with growth factors [158]. Tissue engineering chambers not only play a promising role in soft-tissue reconstruction but also serve as a mechanistic model for understanding tissue growth. While promising, both AVL and vascular bundles still suffer from a lack of rapidity.

#### 3.5.3. Micropuncture

**Figure 6:**
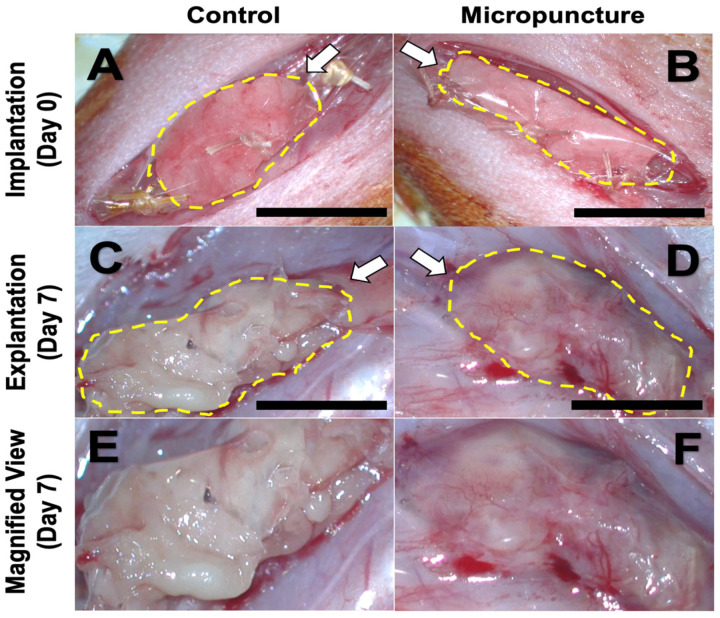
Fat graft vascularization after 7 days. Equal amounts of autologous adipose (yellow outline) was loaded onto a silicone sheet that circumferentially wrapped the femoral vessels (**A**,**B**). After 7 days, FGs were analyzed in situ, with control non-MP fat grafts remaining avascular and undergoing liquefaction necrosis (**C**,**E**) while MP grafts have evidence of robust vessel ingrowth (**D**,**F**). Arrows show the direction of the underlying femoral vessels. Scale bar = 10 mm.

Our group has been using an experimental microsurgery technique termed micropuncture (MP) to stimulate cell extravasation and rapid microvascular formation out of a macrovascular bundle (Figure 5C). Sprouting angiogenesis is a complex and sequential process that requires disruption of the basement membrane. Normally, this is the rate-limiting step in angiogenesis, as there needs to be a substantial buildup of inflammatory cells, MMPs, and cytokines. In microsurgery, we routinely use needles that have diameters in the capillary range, so we sought to explore whether a 60 µm needle could be used to purposely disrupt the vessel basement membrane and rapidly stimulate microvascular outgrowth. To date, we have demonstrated that MPs can expedite adjacent hydrogel scaffold vascularization, with the induced microvasculature demonstrating sustainability for up to one month [159]. Angiogenesis in the MP cohort is induced by increased infiltration of ECs and macrophages and increased expression of VEGF-receptor 2 and Tie-2, which are involved in vascular remodeling [159,160]. We have performed preliminary studies incorporating MPs into the femoral vascular bundle with autologous adipose tissue to determine whether this can improve fat-graft vascularization (Figure 6A,B). In gross analysis, samples undergoing MP demonstrate increased microvasculature formation when compared with the non-MP control (Figure 6C–F). In the next phase, we aim to study the rapid vascularization of an engineered adipose replacement graft with MP.

## 4. Conclusions

Adipose is an abundantly available tissue in the human body with a wide variety of clinical and engineering applications. Soft-tissue reconstructive efforts to date have primarily focused on fat grafting and adipose flaps. Grafts are limited by oxygen diffusion, and are thus only suitable for the reconstruction of small defects, while flaps carry substantial donor-site morbidity. Recently, there have been a multitude of tissue engineering efforts to develop vascularized adipose tissue. Once successful, our landscape of soft-tissue reconstruction will profoundly change for the benefit of patient care.

## Figures and Tables

**Table 1:** Cell types utilized in adipose tissue engineering.

Cell Type	Source	Differentiation Potential	Qualities Relevant to Adipose Engineering
Adipose-derived stem cells (ASCs)	Adipose tissue, stromal vascular fraction (SVF)	Differentiate into adipocytes, endothelial cells, pericyte-like cells	- easy to harvest - low immunogenicity - upregulate vascularization
	Adult somatic cells (e.g., skin fibroblasts)	Potential to differentiate into various cell lines, including endothelial cells	- low immunogenicity - risk of tumorigenicity - high cost - time-consuming to generate and differentiate cells
Human umbilical vein endothelial cells (HUVECs)	Umbilical cord blood vessels	Endothelial cells	- ease of isolation - abundant cell harvest
Endothelial progenitor cells (EPCs)	Circulating blood, bone marrow, umbilical cord blood, adipose tissue	Endothelial cells	- accessible source - low immunogenicity - minimal ethical concerns - limited expansion capacity
Human adipose-derived microvascular endothelial cells (hAMECs)	Adipose tissue, SVF	Endothelial cells	- low cell abundance - mimic the native endothelial cells in adipose tissue - limited expansion capacity

## Data Availability

All content of this article is available and has been shared.

## References

[1] Berry D.C., Stenesen D., Zeve D., Graff J.M. (2013). The developmental origins of adipose tissue. Development.

[2] Lenz M., Arts I.C.W., Peeters R.L.M., de Kok T.M., Ertaylan G. (2020). Adipose tissue in health and disease through the lens of its building blocks. Sci. Rep..

[3] Teresa Minjung O., Chan K., Brennan T., Roden D., Shamouelian D., Chung H.Y., Waner M. (2019). Autologous Fat Grafting Restores Soft-tissue Contour Deformities after Vascular Anomaly Surgery. Plast. Reconstr. Surg. Glob. Open.

[4] Abu-Ghname A., Perdanasari A.T., Reece E.M. (2019). Principles and Applications of Fat Grafting in Plastic Surgery. Semin. Plast. Surg..

[5] Mota de Sa P., Richard A.J., Hang H., Stephens J.M. (2017). Transcriptional Regulation of Adipogenesis. Compr. Physiol..

[6] Farmer S.R. (2006). Transcriptional control of adipocyte formation. Cell Metab..

[7] Siersbaek R., Mandrup S. (2011). Transcriptional networks controlling adipocyte differentiation. Cold Spring Harb. Symp. Quant. Biol..

[8] Rosen E.D., Walkey C.J., Puigserver P., Spiegelman B.M. (2000). Transcriptional regulation of adipogenesis. Genes. Dev..

[9] Tontonoz P., Hu E., Spiegelman B.M. (1994). Stimulation of adipogenesis in fibroblasts by PPAR gamma 2, a lipid-activated transcription factor. Cell.

[10] Barak Y., Nelson M.C., Ong E.S., Jones Y.Z., Ruiz-Lozano P., Chien K.R., Koder A., Evans R.M. (1999). PPAR gamma is required for placental, cardiac, and adipose tissue development. Mol. Cell.

[11] Lefterova M.I., Haakonsson A.K., Lazar M.A., Mandrup S. (2014). PPARgamma and the global map of adipogenesis and beyond. Trends Endocrinol. Metab..

[12] Lee J.E., Ge K. (2014). Transcriptional and epigenetic regulation of PPARgamma expression during adipogenesis. Cell Biosci..

[13] Imai T., Takakuwa R., Marchand S., Dentz E., Bornert J.M., Messaddeq N., Wendling O., Mark M., Desvergne B., Wahli W. (2004). Peroxisome proliferator-activated receptor gamma is required in mature white and brown adipocytes for their survival in the mouse. Proc. Natl. Acad. Sci. USA.

[14] Linhart H.G., Ishimura-Oka K., DeMayo F., Kibe T., Repka D., Poindexter B., Bick R.J., Darlington G.J. (2001). C/EBPalpha is required for differentiation of white, but not brown, adipose tissue. Proc. Natl. Acad. Sci. USA.

[15] Wu Z., Rosen E.D., Brun R., Hauser S., Adelmant G., Troy A.E., McKeon C., Darlington G.J., Spiegelman B.M. (1999). Cross-regulation of C/EBP alpha and PPAR gamma controls the transcriptional pathway of adipogenesis and insulin sensitivity. Mol. Cell.

[16] Tamori Y., Masugi J., Nishino N., Kasuga M. (2002). Role of peroxisome proliferator-activated receptor-gamma in maintenance of the characteristics of mature 3T3-L1 adipocytes. Diabetes.

[17] Wu Z., Bucher N.L., Farmer S.R. (1996). Induction of peroxisome proliferator-activated receptor gamma during the conversion of 3T3 fibroblasts into adipocytes is mediated by C/EBPbeta, C/EBPdelta, and glucocorticoids. Mol. Cell. Biol..

[18] Cao Z., Umek R.M., McKnight S.L. (1991). Regulated expression of three C/EBP isoforms during adipose conversion of 3T3-L1 cells. Genes. Dev..

[19] Lefterova M.I., Zhang Y., Steger D.J., Schupp M., Schug J., Cristancho A., Feng D., Zhuo D., Stoeckert C.J., Liu X.S. (2008). PPARgamma and C/EBP factors orchestrate adipocyte biology via adjacent binding on a genome-wide scale. Genes. Dev..

[20] Kawai T., Autieri M.V., Scalia R. (2021). Adipose tissue inflammation and metabolic dysfunction in obesity. Am. J. Physiol. Cell Physiol..

[21] Fantuzzi G. (2005). Adipose tissue, adipokines, and inflammation. J. Allergy Clin. Immunol..

[22] Han J., Lee J.E., Jin J., Lim J.S., Oh N., Kim K., Chang S.I., Shibuya M., Kim H., Koh G.Y. (2011). The spatiotemporal development of adipose tissue. Development.

[23] Corvera S., Solivan-Rivera J., Yang Loureiro Z. (2022). Angiogenesis in adipose tissue and obesity. Angiogenesis.

[24] Strong A.L., Adidharma W., Brown O.H., Cederna P.S. (2021). Fat Grafting Subjectively Improves Facial Skin Elasticity and Hand Function of Scleroderma Patients. Plast. Reconstr. Surg. Glob. Open.

[25] Pu L.L. (2016). Mechanisms of Fat Graft Survival. Ann. Plast. Surg..

[26] Evans B.G.A., Gronet E.M., Saint-Cyr M.H. (2020). How Fat Grafting Works. Plast. Reconstr. Surg. Glob. Open.

[27] Gan F., Liu L., Zhou Q., Huang W., Huang X., Zhao X. (2022). Effects of adipose-derived stromal cells and endothelial progenitor cells on adipose transplant survival and angiogenesis. PLoS ONE.

[28] Shauly O., Gould D.J., Ghavami A. (2022). Fat Grafting: Basic Science, Techniques, and Patient Management. Plast. Reconstr. Surg. Glob. Open.

[29] Valente D.S., Ely P.B., Kieling L., Konzen A.T., Steffen L.P., Lazzaretti G.S., Zanella R.K. (2024). Breast fat grafting and cancer: A systematic review of the science behind enhancements and concerns. Transl. Breast Cancer Res..

[30] Delaporte T., Delay E., Toussoun G., Delbaere M., Sinna R. (2009). Breast volume reconstruction by lipomodeling technique: About 15 consecutive cases. Ann. Chir. Plast. Esthet..

[31] Khouri R.K., Khouri R.E., Lujan-Hernandez J.R., Khouri K.R., Lancerotto L., Orgill D.P. (2014). Diffusion and perfusion: The keys to fat grafting. Plast. Reconstr. Surg. Glob. Open.

[32] Shih L., Davis M.J., Winocour S.J. (2020). The Science of Fat Grafting. Semin. Plast. Surg..

[33] Strong A.L., Cederna P.S., Rubin J.P., Coleman S.R., Levi B. (2015). The Current State of Fat Grafting: A Review of Harvesting, Processing, and Injection Techniques. Plast. Reconstr. Surg..

[34] Bagheri S.C., Bohluli B., Consky E.K. (2018). Current Techniques in Fat Grafting. Atlas Oral Maxillofac. Surg. Clin. N. Am..

[35] Fuentes-Felix C. (2003). BRAVA: Results did not meet expectations. Aesthet. Surg. J..

[36] Oranges C.M., Striebel J., Tremp M., Madduri S., Kalbermatten D.F., Schaefer D.J. (2018). The Impact of Recipient Site External Expansion in Fat Grafting Surgical Outcomes. Plast. Reconstr. Surg. Glob. Open.

[37] Topcu A., Aydin O.E., Unlu M., Barutcu A., Atabey A. (2012). Increasing the viability of fat grafts by vascular endothelial growth factor. Arch. Facial Plast. Surg..

[38] Gassman A.A., Lewis M.S., Bradley J.P., Lee J.C. (2015). Remote Ischemic Preconditioning Improves the Viability of Donor Lipoaspirate during Murine Fat Transfer. Plast. Reconstr. Surg..

[39] Samdal F., Skolleborg K.C., Berthelsen B. (1992). The effect of preoperative needle abrasion of the recipient site on survival of autologous free fat grafts in rats. Scand. J. Plast. Reconstr. Surg. Hand Surg..

[40] Leong D.T., Hutmacher D.W., Chew F.T., Lim T.C. (2005). Viability and adipogenic potential of human adipose tissue processed cell population obtained from pump-assisted and syringe-assisted liposuction. J. Dermatol. Sci..

[41] Simonacci F., Bertozzi N., Grieco M.P., Grignaffini E., Raposio E. (2017). Procedure, applications, and outcomes of autologous fat grafting. Ann. Med. Surg..

[42] Bauer C.A., Valentino J., Hoffman H.T. (1995). Long-term result of vocal cord augmentation with autogenous fat. Ann. Otol. Rhinol. Laryngol..

[43] Nguyen A., Pasyk K.A., Bouvier T.N., Hassett C.A., Argenta L.C. (1990). Comparative study of survival of autologous adipose tissue taken and transplanted by different techniques. Plast. Reconstr. Surg..

[44] Delay E., Garson S., Tousson G., Sinna R. (2009). Fat injection to the breast: Technique, results, and indications based on 880 procedures over 10 years. Aesthet. Surg. J..

[45] Schipper J.A.M., Tuin A.J., Loonen T.G.J., Dijkstra P.U., Spijkervet F.K.L., Schepers R.H., Jansma J. (2025). Volume and patient satisfaction, 5 years of follow up after facial fat grafting. J. Plast. Reconstr. Aesthet. Surg..

[46] Mehrara B.J., Santoro T.D., Arcilla E., Watson J.P., Shaw W.W., Da Lio A.L. (2006). Complications after microvascular breast reconstruction: Experience with 1195 flaps. Plast. Reconstr. Surg..

[47] Chiu Y.H., Chang D.H., Perng C.K. (2017). Vascular Complications and Free Flap Salvage in Head and Neck Reconstructive Surgery: Analysis of 150 Cases of Reexploration. Ann. Plast. Surg..

[48] Pan X.L., Chen G.X., Shao H.W., Han C.M., Zhang L.P., Zhi L.Z. (2014). Effect of heparin on prevention of flap loss in microsurgical free flap transfer: A meta-analysis. PLoS ONE.

[49] Senchenkov A., Lemaine V., Tran N.V. (2015). Management of perioperative microvascular thrombotic complications—The use of multiagent anticoagulation algorithm in 395 consecutive free flaps. J. Plast. Reconstr. Aesthet. Surg..

[50] Chang E.I., Chang E.I., Soto-Miranda M.A., Zhang H., Nosrati N., Robb G.L., Chang D.W. (2013). Comprehensive analysis of donor-site morbidity in abdominally based free flap breast reconstruction. Plast. Reconstr. Surg..

[51] Atisha D.M., Tessiatore K.M., Rushing C.N., Dayicioglu D., Pusic A., Hwang S. (2019). A National Snapshot of Patient-Reported Outcomes Comparing Types of Abdominal Flaps for Breast Reconstruction. Plast. Reconstr. Surg..

[52] Las D.E., de Jong T., Zuidam J.M., Verweij N.M., Hovius S.E., Mureau M.A. (2016). Identification of independent risk factors for flap failure: A retrospective analysis of 1530 free flaps for breast, head and neck and extremity reconstruction. J. Plast. Reconstr. Aesthet. Surg..

[53] Largo R.D., Selber J.C., Garvey P.B., Chang E.I., Hanasono M.M., Yu P., Butler C.E., Baumann D.P. (2018). Outcome Analysis of Free Flap Salvage in Outpatients Presenting with Microvascular Compromise. Plast. Reconstr. Surg..

[54] Langer R., Vacanti J.P. (1993). Tissue engineering. Science.

[55] Peirsman A., Nguyen H.T., Van Waeyenberge M., Ceballos C., Bolivar J., Kawakita S., Vanlauwe F., Tirpakova Z., Van Dorpe S., Van Damme L. (2023). Vascularized adipose tissue engineering: Moving towards soft tissue reconstruction. Biofabrication.

[56] Bianco P. (2014). “Mesenchymal” stem cells. Annu. Rev. Cell Dev. Biol..

[57] Si Z., Wang X., Sun C., Kang Y., Xu J., Wang X., Hui Y. (2019). Adipose-derived stem cells: Sources, potency, and implications for regenerative therapies. Biomed. Pharmacother..

[58] Bacakova L., Zarubova J., Travnickova M., Musilkova J., Pajorova J., Slepicka P., Kasalkova N.S., Svorcik V., Kolska Z., Motarjemi H. (2018). Stem cells: Their source, potency and use in regenerative therapies with focus on adipose-derived stem cells—A review. Biotechnol. Adv..

[59] Huang Q., Zou Y., Arno M.C., Chen S., Wang T., Gao J., Dove A.P., Du J. (2017). Hydrogel scaffolds for differentiation of adipose-derived stem cells. Chem. Soc. Rev..

[60] Liu Y., Liu Y., Wu M., Zou R., Mao S., Cong P., Hou M., Jin H., Zhao Y., Bao Y. (2022). Adipose-derived mesenchymal stem cell-loaded beta-chitin nanofiber hydrogel promote wound healing in rats. J. Mater. Sci. Mater. Med..

[61] Pacelli S., Chakravarti A.R., Modaresi S., Subham S., Burkey K., Kurlbaum C., Fang M., Neal C.A., Mellott A.J., Chakraborty A. (2021). Investigation of human adipose-derived stem-cell behavior using a cell-instructive polydopamine-coated gelatin-alginate hydrogel. J. Biomed. Mater. Res. A.

[62] Razavi S., Karbasi S., Morshed M., Zarkesh Esfahani H., Golozar M., Vaezifar S. (2015). Cell Attachment and Proliferation of Human Adipose-Derived Stem Cells on PLGA/Chitosan Electrospun Nano-Biocomposite. Cell J..

[63] Yang J.Z., Qiu L.H., Xiong S.H., Dang J.L., Rong X.K., Hou M.M., Wang K., Yu Z., Yi C.G. (2020). Decellularized adipose matrix provides an inductive microenvironment for stem cells in tissue regeneration. World J. Stem Cells.

[64] Hong K.Y., Bae H., Park I., Park D.Y., Kim K.H., Kubota Y., Cho E.S., Kim H., Adams R.H., Yoo O.J. (2015). Perilipin+ embryonic preadipocytes actively proliferate along growing vasculatures for adipose expansion. Development.

[65] Tsekouras A., Mantas D., Tsilimigras D.I., Moris D., Kontos M., Zografos G.C. (2017). Comparison of the Viability and Yield of Adipose-Derived Stem Cells (ASCs) from Different Donor Areas. In Vivo.

[66] Dani V., Yao X., Dani C. (2022). Transplantation of fat tissues and iPSC-derived energy expenditure adipocytes to counteract obesity-driven metabolic disorders: Current strategies and future perspectives. Rev. Endocr. Metab. Disord..

[67] Patsch C., Challet-Meylan L., Thoma E.C., Urich E., Heckel T., O’Sullivan J.F., Grainger S.J., Kapp F.G., Sun L., Christensen K. (2015). Generation of vascular endothelial and smooth muscle cells from human pluripotent stem cells. Nat. Cell Biol..

[68] Kong A.M., Yap K.K., Lim S.Y., Marre D., Pebay A., Gerrand Y.W., Lees J.G., Palmer J.A., Morrison W.A., Mitchell G.M. (2019). Bio-engineering a tissue flap utilizing a porous scaffold incorporating a human induced pluripotent stem cell-derived endothelial cell capillary network connected to a vascular pedicle. Acta Biomater..

[69] Koduru S.V., Leberfinger A.N., Pasic D., Forghani A., Lince S., Hayes D.J., Ozbolat I.T., Ravnic D.J. (2019). Cellular Based Strategies for Microvascular Engineering. Stem Cell Rev. Rep..

[70] Koivunotko E., Snirvi J., Merivaara A., Harjumaki R., Rautiainen S., Kelloniemi M., Kuismanen K., Miettinen S., Yliperttula M., Koivuniemi R. (2022). Angiogenic Potential of Human Adipose-Derived Mesenchymal Stromal Cells in Nanofibrillated Cellulose Hydrogel. Biomedicines.

[71] Sarkanen J.R., Vuorenpaa H., Huttala O., Mannerstrom B., Kuokkanen H., Miettinen S., Heinonen T., Ylikomi T. (2012). Adipose stromal cell tubule network model provides a versatile tool for vascular research and tissue engineering. Cells Tissues Organs.

[72] Zhang Q., Johnson J.A., Dunne L.W., Chen Y., Iyyanki T., Wu Y., Chang E.I., Branch-Brooks C.D., Robb G.L., Butler C.E. (2016). Decellularized skin/adipose tissue flap matrix for engineering vascularized composite soft tissue flaps. Acta Biomater..

[73] Kocherova I., Bryja A., Mozdziak P., Angelova Volponi A., Dyszkiewicz-Konwinska M., Piotrowska-Kempisty H., Antosik P., Bukowska D., Bruska M., Izycki D. (2019). Human Umbilical Vein Endothelial Cells (HUVECs) Co-Culture with Osteogenic Cells: From Molecular Communication to Engineering Prevascularised Bone Grafts. J. Clin. Med..

[74] Prasad Chennazhy K., Krishnan L.K. (2005). Effect of passage number and matrix characteristics on differentiation of endothelial cells cultured for tissue engineering. Biomaterials.

[75] Yoder M.C. (2012). Human endothelial progenitor cells. Cold Spring Harb. Perspect. Med..

[76] Freiman A., Shandalov Y., Rozenfeld D., Shor E., Segal S., Ben-David D., Meretzki S., Egozi D., Levenberg S. (2016). Adipose-derived endothelial and mesenchymal stem cells enhance vascular network formation on three-dimensional constructs in vitro. Stem Cell Res. Ther..

[77] Yue B. (2014). Biology of the extracellular matrix: An overview. J. Glaucoma.

[78] Frantz C., Stewart K.M., Weaver V.M. (2010). The extracellular matrix at a glance. J. Cell Sci..

[79] Fan L., Ren Y., Emmert S., Vuckovic I., Stojanovic S., Najman S., Schnettler R., Barbeck M., Schenke-Layland K., Xiong X. (2023). The Use of Collagen-Based Materials in Bone Tissue Engineering. Int. J. Mol. Sci..

[80] Kimura Y., Inamoto T., Tabata Y. (2010). Adipose tissue formation in collagen scaffolds with different biodegradabilities. J. Biomater. Sci. Polym. Ed..

[81] Kimura Y., Ozeki M., Inamoto T., Tabata Y. (2003). Adipose tissue engineering based on human preadipocytes combined with gelatin microspheres containing basic fibroblast growth factor. Biomaterials.

[82] Yang G., Xiao Z., Long H., Ma K., Zhang J., Ren X., Zhang J. (2018). Assessment of the characteristics and biocompatibility of gelatin sponge scaffolds prepared by various crosslinking methods. Sci. Rep..

[83] Davidenko N., Campbell J.J., Thian E.S., Watson C.J., Cameron R.E. (2010). Collagen-hyaluronic acid scaffolds for adipose tissue engineering. Acta Biomater..

[84] Zhu Y., Liu T., Song K., Jiang B., Ma X., Cui Z. (2009). Collagen-chitosan polymer as a scaffold for the proliferation of human adipose tissue-derived stem cells. J. Mater. Sci. Mater. Med..

[85] Albrecht F.B., Schmidt F.F., Volz A.C., Kluger P.J. (2022). Bioprinting of 3D Adipose Tissue Models Using a GelMA-Bioink with Human Mature Adipocytes or Human Adipose-Derived Stem Cells. Gels.

[86] Cheng M.H., Chang C.W., Wang J., Bupphathong S., Huang W., Lin C.H. (2024). 3D-Bioprinted GelMA Scaffold with ASCs and HUVECs for Engineering Vascularized Adipose Tissue. ACS Appl. Bio Mater..

[87] Gilchrist A.E., Serrano J.F., Ngo M.T., Hrnjak Z., Kim S., Harley B.A.C. (2021). Encapsulation of murine hematopoietic stem and progenitor cells in a thiol-crosslinked maleimide-functionalized gelatin hydrogel. Acta Biomater..

[88] Chang K.H., Liao H.T., Chen J.P. (2013). Preparation and characterization of gelatin/hyaluronic acid cryogels for adipose tissue engineering: In vitro and in vivo studies. Acta Biomater..

[89] Janmey P.A., Winer J.P., Weisel J.W. (2009). Fibrin gels and their clinical and bioengineering applications. J. R. Soc. Interface.

[90] Wittmann K., Dietl S., Ludwig N., Berberich O., Hoefner C., Storck K., Blunk T., Bauer-Kreisel P. (2015). Engineering vascularized adipose tissue using the stromal-vascular fraction and fibrin hydrogels. Tissue Eng. Part. A.

[91] Holnthoner W., Hohenegger K., Husa A.M., Muehleder S., Meinl A., Peterbauer-Scherb A., Redl H. (2015). Adipose-derived stem cells induce vascular tube formation of outgrowth endothelial cells in a fibrin matrix. J. Tissue Eng. Regen. Med..

[92] Ahmed T.A., Griffith M., Hincke M. (2007). Characterization and inhibition of fibrin hydrogel-degrading enzymes during development of tissue engineering scaffolds. Tissue Eng..

[93] Louis F., Sowa Y., Irie S., Higuchi Y., Kitano S., Mazda O., Matsusaki M. (2022). Injectable Prevascularized Mature Adipose Tissues (iPAT) to Achieve Long-Term Survival in Soft Tissue Regeneration. Adv. Healthc. Mater..

[94] Lee K.Y., Mooney D.J. (2012). Alginate: Properties and biomedical applications. Prog. Polym. Sci..

[95] Kang S.W., Cha B.H., Park H., Park K.S., Lee K.Y., Lee S.H. (2011). The effect of conjugating RGD into 3D alginate hydrogels on adipogenic differentiation of human adipose-derived stromal cells. Macromol. Biosci..

[96] Yoo B., Kim S., Shin B.H., Lee M.H., Choy Y.B., Lee K., Heo C.Y., Koh W.G. (2021). Preparation of alginate hydrogel with human-derived adipose tissue to improve fat graft survival and adipogenesis. J. Ind. Eng. Chem..

[97] Kim W.S., Mooney D.J., Arany P.R., Lee K., Huebsch N., Kim J. (2012). Adipose tissue engineering using injectable, oxidized alginate hydrogels. Tissue Eng. Part. A.

[98] Brandl F.P., Seitz A.K., Tessmar J.K., Blunk T., Gopferich A.M. (2010). Enzymatically degradable poly(ethylene glycol) based hydrogels for adipose tissue engineering. Biomaterials.

[99] Salvatore L., Natali M.L., Brunetti C., Sannino A., Gallo N. (2023). An Update on the Clinical Efficacy and Safety of Collagen Injectables for Aesthetic and Regenerative Medicine Applications. Polymers.

[100] Van Nieuwenhove I., Tytgat L., Ryx M., Blondeel P., Stillaert F., Thienpont H., Ottevaere H., Dubruel P., Van Vlierberghe S. (2017). Soft tissue fillers for adipose tissue regeneration: From hydrogel development toward clinical applications. Acta Biomater..

[101] Vashi A.V., Keramidaris E., Abberton K.M., Morrison W.A., Wilson J.L., O’Connor A.J., Cooper-White J.J., Thompson E.W. (2008). Adipose differentiation of bone marrow-derived mesenchymal stem cells using Pluronic F-127 hydrogel in vitro. Biomaterials.

[102] Weiser B., Prantl L., Schubert T.E., Zellner J., Fischbach-Teschl C., Spruss T., Seitz A.K., Tessmar J., Goepferich A., Blunk T. (2008). In vivo development and long-term survival of engineered adipose tissue depend on in vitro precultivation strategy. Tissue Eng. Part. A.

[103] Xu J., Chen Y., Yue Y., Sun J., Cui L. (2012). Reconstruction of epidural fat with engineered adipose tissue from adipose derived stem cells and PLGA in the rabbit dorsal laminectomy model. Biomaterials.

[104] Patrick C.W., Chauvin P.B., Hobley J., Reece G.P. (1999). Preadipocyte seeded PLGA scaffolds for adipose tissue engineering. Tissue Eng..

[105] Fukushima K. (2016). Poly(trimethylene carbonate)-based polymers engineered for biodegradable functional biomaterials. Biomater. Sci..

[106] Zhang Z., Kuijer R., Bulstra S.K., Grijpma D.W., Feijen J. (2006). The in vivo and in vitro degradation behavior of poly(trimethylene carbonate). Biomaterials.

[107] Jain S., Yassin M.A., Fuoco T., Liu H., Mohamed-Ahmed S., Mustafa K., Finne-Wistrand A. (2020). Engineering 3D degradable, pliable scaffolds toward adipose tissue regeneration; optimized printability, simulations and surface modification. J. Tissue Eng..

[108] Chiu Y.C., Cheng M.H., Engel H., Kao S.W., Larson J.C., Gupta S., Brey E.M. (2011). The role of pore size on vascularization and tissue remodeling in PEG hydrogels. Biomaterials.

[109] Lee S., Lee H.S., Chung J.J., Kim S.H., Park J.W., Lee K., Jung Y. (2021). Enhanced Regeneration of Vascularized Adipose Tissue with Dual 3D-Printed Elastic Polymer/dECM Hydrogel Complex. Int. J. Mol. Sci..

[110] Banyard D.A., Borad V., Amezcua E., Wirth G.A., Evans G.R., Widgerow A.D. (2016). Preparation, Characterization, and Clinical Implications of Human Decellularized Adipose Tissue Extracellular Matrix (hDAM): A Comprehensive Review. Aesthet. Surg. J..

[111] Wang L., Johnson J.A., Zhang Q., Beahm E.K. (2013). Combining decellularized human adipose tissue extracellular matrix and adipose-derived stem cells for adipose tissue engineering. Acta Biomater..

[112] Flynn L.E. (2010). The use of decellularized adipose tissue to provide an inductive microenvironment for the adipogenic differentiation of human adipose-derived stem cells. Biomaterials.

[113] Han T.T., Toutounji S., Amsden B.G., Flynn L.E. (2015). Adipose-derived stromal cells mediate in vivo adipogenesis, angiogenesis and inflammation in decellularized adipose tissue bioscaffolds. Biomaterials.

[114] Cheung H.K., Han T.T., Marecak D.M., Watkins J.F., Amsden B.G., Flynn L.E. (2014). Composite hydrogel scaffolds incorporating decellularized adipose tissue for soft tissue engineering with adipose-derived stem cells. Biomaterials.

[115] Choi J.S., Kim B.S., Kim J.Y., Kim J.D., Choi Y.C., Yang H.J., Park K., Lee H.Y., Cho Y.W. (2011). Decellularized extracellular matrix derived from human adipose tissue as a potential scaffold for allograft tissue engineering. J. Biomed. Mater. Res. A.

[116] Pati F., Ha D.H., Jang J., Han H.H., Rhie J.W., Cho D.W. (2015). Biomimetic 3D tissue printing for soft tissue regeneration. Biomaterials.

[117] Flynn L., Semple J.L., Woodhouse K.A. (2006). Decellularized placental matrices for adipose tissue engineering. J. Biomed. Mater. Res. A.

[118] Xu M., He Y., Li Y., Liu K., Zhang Y., Su T., Yao Y., Jin X., Zhang X., Lu F. (2024). Combined Use of Autologous Sustained-Release Scaffold of Adipokines and Acellular Adipose Matrix to Construct Vascularized Adipose Tissue. Plast. Reconstr. Surg..

[119] Lee E.Y., Xia Y., Kim W.S., Kim M.H., Kim T.H., Kim K.J., Park B.S., Sung J.H. (2009). Hypoxia-enhanced wound-healing function of adipose-derived stem cells: Increase in stem cell proliferation and up-regulation of VEGF and bFGF. Wound Repair. Regen..

[120] Irvin J., Danchik C., Rall J., Babcock A., Pine M., Barnaby D., Pathakamuri J., Kuebler D. (2018). Bioactivity and composition of a preserved connective tissue matrix derived from human placental tissue. J. Biomed. Mater. Res. B Appl. Biomater..

[121] Magana A., Giovanni R., Essien E., Epel B., Kotecha M., Liu S., Mathew M.T., Hagarty S.E., Bijukumar D. (2022). Amniotic growth factors enhanced human pre-adipocyte cell viability and differentiation under hypoxia. J. Biomed. Mater. Res. B Appl. Biomater..

[122] Ikegami Y., Mizumachi H., Yoshida K., Ijima H. (2020). Heparin-conjugated collagen as a potent growth factor-localizing and stabilizing scaffold for regenerative medicine. Regen. Ther..

[123] Khanna A., Oropeza B.P., Huang N.F. (2022). Engineering Spatiotemporal Control in Vascularized Tissues. Bioengineering.

[124] Song M., Zhou Y., Liu Y. (2018). VEGF heparinized-decellularized adipose tissue scaffolds enhance tissue engineering vascularization in vitro. RSC Adv..

[125] Murohara T., Shintani S., Kondo K. (2009). Autologous adipose-derived regenerative cells for therapeutic angiogenesis. Curr. Pharm. Des..

[126] Todorova D., Simoncini S., Lacroix R., Sabatier F., Dignat-George F. (2017). Extracellular Vesicles in Angiogenesis. Circ. Res..

[127] Ju Y., Hu Y., Yang P., Xie X., Fang B. (2023). Extracellular vesicle-loaded hydrogels for tissue repair and regeneration. Mater. Today Bio.

[128] Mou S., Zhou M., Li Y., Wang J., Yuan Q., Xiao P., Sun J., Wang Z. (2019). Extracellular Vesicles from Human Adipose-Derived Stem Cells for the Improvement of Angiogenesis and Fat-Grafting Application. Plast. Reconstr. Surg..

[129] Nie F., Ding P., Zhang C., Zhao Z., Bi H. (2021). Extracellular vesicles derived from lipoaspirate fluid promote fat graft survival. Adipocyte.

[130] Zhang Y., Liu T. (2023). Adipose-derived stem cells exosome and its potential applications in autologous fat grafting. J. Plast. Reconstr. Aesthet. Surg..

[131] Yang S., Jiang H., Qian M., Ji G., Wei Y., He J., Tian H., Zhao Q. (2022). MSC-derived sEV-loaded hyaluronan hydrogel promotes scarless skin healing by immunomodulation in a large skin wound model. Biomed. Mater..

[132] Vyas K.S., Vasconez H.C., Morrison S., Mogni B., Linton S., Hockensmith L., Kabir T., Zielins E., Najor A., Bakri K. (2020). Fat Graft Enrichment Strategies: A Systematic Review. Plast. Reconstr. Surg..

[133] Li J., Shi X., Chen W. (2013). Influence of repeatedly injecting platelet-rich plasma on survival and quality of fat grafts in nude mice. Zhongguo Xiu Fu Chong Jian Wai Ke Za Zhi.

[134] Sasaki G.H. (2015). The Safety and Efficacy of Cell-Assisted Fat Grafting to Traditional Fat Grafting in the Anterior Mid-Face: An Indirect Assessment by 3D Imaging. Aesthetic Plast. Surg..

[135] Gentile P., Di Pasquali C., Bocchini I., Floris M., Eleonora T., Fiaschetti V., Floris R., Cervelli V. (2013). Breast reconstruction with autologous fat graft mixed with platelet-rich plasma. Surg. Innov..

[136] Lu T., Li Y., Chen T. (2013). Techniques for fabrication and construction of three-dimensional scaffolds for tissue engineering. Int. J. Nanomedicine.

[137] Shimizu T. (2014). Cell sheet-based tissue engineering for fabricating 3-dimensional heart tissues. Circ. J..

[138] Villalona G.A., Udelsman B., Duncan D.R., McGillicuddy E., Sawh-Martinez R.F., Hibino N., Painter C., Mirensky T., Erickson B., Shinoka T. (2010). Cell-seeding techniques in vascular tissue engineering. Tissue Eng. Part. B Rev..

[139] Nichol J.W., Khademhosseini A. (2009). Modular Tissue Engineering: Engineering Biological Tissues from the Bottom Up. Soft Matter.

[140] Brett E., Chung N., Leavitt W.T., Momeni A., Longaker M.T., Wan D.C. (2017). A Review of Cell-Based Strategies for Soft Tissue Reconstruction. Tissue Eng. Part. B Rev..

[141] Zhang Q., Hubenak J., Iyyanki T., Alred E., Turza K.C., Davis G., Chang E.I., Branch-Brooks C.D., Beahm E.K., Butler C.E. (2015). Engineering vascularized soft tissue flaps in an animal model using human adipose-derived stem cells and VEGF+PLGA/PEG microspheres on a collagen-chitosan scaffold with a flow-through vascular pedicle. Biomaterials.

[142] Laschke M.W., Menger M.D. (2017). Spheroids as vascularization units: From angiogenesis research to tissue engineering applications. Biotechnol. Adv..

[143] Di Stefano A.B., Urrata V., Trapani M., Moschella F., Cordova A., Toia F. (2022). Systematic review on spheroids from adipose-derived stem cells: Spontaneous or artefact state?. J. Cell. Physiol..

[144] Banerjee D., Singh Y.P., Datta P., Ozbolat V., O’Donnell A., Yeo M., Ozbolat I.T. (2022). Strategies for 3D bioprinting of spheroids: A comprehensive review. Biomaterials.

[145] He J., Zhang X., Xia X., Han M., Li F., Li C., Li Y., Gao D. (2020). Organoid technology for tissue engineering. J. Mol. Cell Biol..

[146] Mandl M., Viertler H.P., Hatzmann F.M., Brucker C., Grossmann S., Waldegger P., Rauchenwald T., Mattesich M., Zwierzina M., Pierer G. (2022). An organoid model derived from human adipose stem/progenitor cells to study adipose tissue physiology. Adipocyte.

[147] Strobel H.A., Gerton T., Hoying J.B. (2021). Vascularized adipocyte organoid model using isolated human microvessel fragments. Biofabrication.

[148] Schmidt V.J., Hilgert J.G., Covi J.M., Leibig N., Wietbrock J.O., Arkudas A., Polykandriotis E., de Wit C., Horch R.E., Kneser U. (2015). Flow increase is decisive to initiate angiogenesis in veins exposed to altered hemodynamics. PLoS ONE.

[149] Leibig N., Wietbrock J.O., Bigdeli A.K., Horch R.E., Kremer T., Kneser U., Schmidt V.J. (2016). Flow-Induced Axial Vascularization: The Arteriovenous Loop in Angiogenesis and Tissue Engineering. Plast. Reconstr. Surg..

[150] Fischer K.S., Henn D., Zhao E.T., Sivaraj D., Litmanovich B., Hahn W.W., Hostler A.C., Mojadidi S.M., Gonzalez J., Knochel A.B. (2024). Elevated Shear Stress Modulates Heterogenous Cellular Subpopulations to Induce Vascular Remodeling. Tissue Eng. Part. A.

[151] Schmidt V.J., Hilgert J.G., Covi J.M., Weis C., Wietbrock J.O., de Wit C., Horch R.E., Kneser U. (2013). High flow conditions increase connexin43 expression in a rat arteriovenous and angioinductive loop model. PLoS ONE.

[152] Meyer A., Horch R.E., Schoengart E., Beier J.P., Taeger C.D., Arkudas A., Lang W. (2016). Results of combined vascular reconstruction by means of AV loops and free flap transfer in patients with soft tissue defects. J. Plast. Reconstr. Aesthet. Surg..

[153] Debels H., Palmer J., Han X.L., Poon C., Abberton K., Morrison W. (2020). In vivo tissue engineering of an adipose tissue flap using fat grafts and Adipogel. J. Tissue Eng. Regen. Med..

[154] Henn D., Chen K., Fischer K., Rauh A., Barrera J.A., Kim Y.J., Martin R.A., Hannig M., Niedoba P., Reddy S.K. (2020). Tissue Engineering of Axially Vascularized Soft-Tissue Flaps with a Poly-(varepsilon-Caprolactone) Nanofiber-Hydrogel Composite. Adv. Wound Care.

[155] Rnjak-Kovacina J., Gerrand Y.W., Wray L.S., Tan B., Joukhdar H., Kaplan D.L., Morrison W.A., Mitchell G.M. (2019). Vascular Pedicle and Microchannels: Simple Methods Toward Effective In Vivo Vascularization of 3D Scaffolds. Adv. Healthc. Mater..

[156] Tanaka Y., Sung K.C., Fumimoto M., Tsutsumi A., Kondo S., Hinohara Y., Morrison W.A. (2006). Prefabricated engineered skin flap using an arteriovenous vascular bundle as a vascular carrier in rabbits. Plast. Reconstr. Surg..

[157] Tanaka Y., Tamai M., Taguchi N., Niyazi A., Ueno M., Nagasao T. (2020). Spontaneously generated large adipose flaps in vivo tissue engineering chambers. J. Plast. Reconstr. Aesthet. Surg..

[158] Lu Z., Yuan Y., Gao J., Lu F. (2016). Adipose tissue extract promotes adipose tissue regeneration in an adipose tissue engineering chamber model. Cell Tissue Res..

[159] Horchler S.N., Hancock P.C., Sun M., Liu A.T., Massand S., El-Mallah J.C., Goldenberg D., Waldron O., Landmesser M.E., Agrawal S. (2024). Vascular persistence following precision micropuncture. Microcirculation.

[160] Hancock P.C., Koduru S.V., Sun M., Ravnic D.J. (2021). Induction of scaffold angiogenesis by recipient vasculature precision micropuncture. Microvasc. Res..

